# IL-1β and iNOS can drive the asthmatic comorbidities and decrease of lung function in perennial allergic rhinitis children

**DOI:** 10.1186/s13223-023-00867-3

**Published:** 2024-01-02

**Authors:** Myung Woul Han, Song Hee Kim, Inbo Oh, Yang ho Kim, Jiho Lee

**Affiliations:** 1 Department of Otolaryngology, Ulsan University Hospital, University of Ulsan College of Medicine, Ulsan, Republic of Korea; 2 Environmental Health Center, University of Ulsan College of Medicine, Ulsan, Republic of Korea; 3 Department of Occupational and Environmental Medicine, Ulsan University Hospital, University of Ulsan College of Medicine, Ulsan, Republic of Korea

**Keywords:** Allergic rhinitis, Asthma, Comorbidity, IL-1β, iNOS, Epithelial-mesenchymal transition, E-cadherin, Vimentin

## Abstract

**Background:**

Allergic asthma and rhinitis (AR) are closely linked, with a significant proportion of AR patients developing asthma. Identification of the early signs of comorbidity of AR and asthma can enable prompt treatment and prevent asthma progression.

**Objectives and methods:**

This study investigated the role of interleukin-1β (IL-1β), a pro-inflammatory cytokine, and inducible nitric oxide synthase (iNOS) in the comorbidity of AR and asthma and lung function in Korean children with perennial AR (PAR). A cohort of 240 subjects (6 to 10 years old) with PAR (PAR alone: 113 children, PAR and asthma: 127 children) was analyzed for various biomarkers, including IL-1β, iNOS, and epithelial-mesenchymal transition (EMT) markers in serum. The blood levels of eosinophils and immunoglobulin E (IgE) were examined. IL-1β, CCL-24, E-cadherin, and vimentin were measured by enzyme-linked immunosorbent assay (ELISA). Epithelial iNOS was measured by the NOS kit.

**Results:**

Elevated levels of IL-1β, iNOS, and vimentin in the serum were identified as significant indicators of the likelihood of comorbidity of PAR and asthma in children. Furthermore, higher concentrations of IL-1β, iNOS, and vimentin have been linked to reduced lung function in PAR children. Notably, IL-1β expression shows a relationship with the levels of E-cadherin, vimentin, and CCL-24. However, no correlation was found between IL-1β and iNOS expressions.

**Conclusions:**

This study suggests that IL-1β and iNOS can be biomarkers in the progression of PAR and asthma and decreased lung function, suggesting potential targets for early intervention and treatment.

## Introduction

Allergic asthma and rhinitis (AR) are epidemiologically and biologically linked, and 30% of AR develop asthma, and 80% of patients with perennial asthma have AR. Many children with AR have asthma, and several studies have supported that AR is a risk factor for developing asthma [1]. Uncontrolled severe AR can exacerbate asthma in children. The therapy of AR such as antihistamines, and topical intranasal corticosteroids can improve AR symptoms with concomitant asthma, and immunotherapy offers prevention of developing asthma [2–7]. Therefore, the identification of the early signs of comorbidity of AR and asthma can enable prompt treatment and prevent disease progression. Although the Allergic Rhinitis and its Impact on Asthma (ARIA) report have ongoing studies to identify risk factors or protective factors in patients with multimorbidity, sufficient evidence has not been obtained [8].

In the respiratory epithelium, nitric oxide (NO) is a crucial regulator of various physiological functions of the airway epithelium. It is synthesized from arginine by the action of NO synthase (NOS), which comprises constitutive neuronal NOS (nNOS), inducible NOS (iNOS), and constitutive endothelial or epithelial NOS (eNOS). Specifically, pro-inflammatory stimuli and cytokines induce epithelial iNOS expression, increasing NO, and IL-1β and TNF-α are involved in the synthesis of tetrahydrobiopterin (BH4), an essential cofactor for iNOS activity [9]. Increased NO concentration by dysregulation of iNOS activity induces chronic inflammatory responses and nitration of proteins that trigger bronchial epithelial tissue injury that leads to various pulmonary diseases such as asthma [9].

Airway remodeling plays a vital role in increased airway hyperresponsiveness, airflow obstruction, and irreversible decrease in lung function in asthma and is considered the result of repetitive injury caused by chronic airway inflammation [10]. Epithelial-mesenchymal transition (EMT) is a pathophysiological process induced by multiple signaling pathways, including a decrease in E-cadherin and an increase in N-cadherin or vimentin expression [11]. Previous studies have shown that various inflammatory factors, including innate immune cytokines and type 2 cytokines, are involved in EMT, may contribute to airway remodeling, and lead to poor asthma control [12, 13]. The understanding of EMT’s role in asthma remains ambiguous due to the complex nature of EMT and the limited number of in vivo studies conducted [10, 11].

In previous studies, we reported that the activation of IL-1β, a pro-inflammatory cytokine, might be a risk factor for moderate perennial AR and worsening of AR symptoms [14]. Therefore, this study investigated the role of IL-1β, which has been demonstrated to be an important marker in previous studies [14], in the progression of comorbid disease and decline in lung function, along with the expression of various biomarkers in Korean children with PAR.

## Patients and methods

### Study participants

This study included children enrolled in the Elementary School Student Cohort (2009–2016) to identify various factors of allergic disease at Environmental Health Center of Ulsan University Hospital (Ulsan, Korea) [15]. This study was approved by the institutional ethics review committee of the Ulsan University Hospital (approval number 2009-09-061-011). A total of 240 participants (6 to 10 years old) with PAR (PAR alone: 113 children, PAR and asthma: 127 children) were recruited in a nested case-control study. We included the only PAR children, having an inflammatory process all year round. The group of PAR and asthma was included as children with asthmatic symptoms for one year. Subjects received a detailed questionnaire at enrollment and laboratory tests were performed. All students responded to the questionnaire and underwent routine medical checks, including a skin prick test, a blood test for total IgE levels and eosinophil counts, and a lung function test. The questionnaires used in this study included survey questions from the International Study of Asthma and Allergies in Children [16] and questions regarding the socioeconomic status of the patients and hazardous environmental factors. Parents or guardians of all participants provided their written informed consent. We assembled a cohort of patients diagnosed with perennial AR to conduct a nested case-control study. We identified all children who were diagnosed with AR based on clinical symptoms and objective IgE-mediated skin prick tests. The diagnosis of asthma was determined according to the Korean Guideline for Asthma 2021 and was based on a history of variable respiratory symptoms (e.g., wheezing, shortness of breath, chest tightness and cough) and variable expiratory airflow limitation. At a time when FEV1 is reduced, confirm that FEV1/FVC is reduced compared with the lower limit of normal (it is usually > 0.8 in children) [17]. A positive bronchodilator responsiveness (reversibility) test was conducted in case of a history of respiratory symptoms and an increase in FEV1 by > 12% predicted was confident the diagnosis before and 15 min after inhalation of 200 µg salbutamol. Participants are usually asked to withhold bronchodilator medications for 12 h before the test [17, 18].

### Diagnosis of AR

We used the clinical definition of AR for diagnosis, which includes AR symptoms such as rhinorrhea, nasal obstruction, nasal itching, and sneezing, which are reversible spontaneously or with treatment, and objective tests of IgE-mediated allergies, such as the skin prick test. As part of the study, the skin prick test was performed during the medical examination to measure responses to 18 allergens (*Dermatophagoides pteronyssinus, Dermatophagoides farinae*, willow, birch, plantain, grass pollen, alder, Oak, ragweed, mugwort, cat fur, dog fur, cockroach, *Aspergillus, Alternaria* species, and other fungus mixtures, milk, egg) compared with the positive and negative control. Positive reactions for each allergen were defined as a wheal diameter at least 3 mm greater than that of the negative control.

### Pulmonary function test (PFT)

Spirometry (Microspiro HI-801, Tokyo, Japan) was used to measure lung function. Before measurement, the child was fully instructed how to perform the test, stabilized, and then asked to bite the mouthpiece in a standing position, inhale as deeply as possible, and exhale as hard and fast as possible for at least 6 s to obtain a flow-volume curve. Forced vital capacity (FVC), forced expiratory volume in one second (FEV1) and forced expiratory flow at 25–75% of FVC (FEF 25–75%) were measured in triplicate and the highest measurement was taken. The test was performed according to the American Thoracic Society standard test and the determination of decreased lung function was based on the study by Park et al. (Normalized spirometric values in Korean children) [19].

### Measurement and analysis of serum biomarkers

To determine the expression of inflammatory or airway remodeling related biomarkers in children with perennial AR, expression level of several biomarkers was measured in the serum. Blood levels of eosinophils and immunoglobulin E (IgE) were examined. Serum IL-1β (which represents the activation of inflammasomes), CCL-24 (chemokines that induce eosinophils in allergic diseases) and the EMT marker (E-cadherin, vimentin) were measured by enzyme-linked immunosorbent assay (ELISA). In addition, epithelial iNOS data were measured by a NOS kit (**Cat. No.** ELH-ENOS). The details of each of the ELISA kits used in the study are as follows: IL-1β (catalog No. DLB50), CCL-24 (catalog No. DCC240B), E-cadherin (ab233611), and vimentin (KA3127, Abnova). Kits were obtained from R & D Systems (Minneapolis, MN), Abcam (Cambridge, MA), and Novus Biologicals (Centennial, CO).

### Statistical analysis

Independent *t-*tests were used to analyze baseline differences using geometric mean after log transformation (ln) and 95% confidence interval. Furthermore, univariate and multivariate analyses were performed to determine possible relationships between variables and the development of comorbidity and decreased lung function. Multiple logistic regression analyses were performed using significant variables in the univariate analysis and were reported to be associated with the development of severe AR. A *P-*value less than 0.05 was considered statistically significant in all analyses. Analyses of the receiver operating characteristic [20] curve and the differences in the area under curves [21] were used to estimate the diagnostic precision of each test. We also determined whether IL-1β, CCL24, iNOS, E-cadherin, and vimentin could be used diagnostically in children with comorbidity of PAR and asthma with classification according to the cut-off value in the ROC curves. The cut-off values of IL-1β, CCL24, iNOS, E-cadherin, and vimentin for comorbidity of PAR and asthma were 10.84 pg/ml, 471.01 pg/ml, 43.63 pg/ml, 93.39 pg/ml, and 486.0 pg/ml.

## Results

### Characteristics of participants and comparison of PAR alone and PAR and asthma group

The characteristics of 113 participants (Male: Female = 63:50, mean age 7.6 years) with PAR alone and 127 participants (Male: Female = 70:57, mean age 7.8 years) with PAR and asthma in this study are summarized in Table 1. We compared the biomarker expression level using geometric mean. There were no significant differences between the PAR alone group and the PAR and asthma group in total IgE, eosinophil count and vimentin. IL-1β, CCL24, and iNOS were significantly higher and E-cadherin was lower in the PAR and asthma group (Table 1). Additionally, there was no difference in the sensitization pattern of skin tests between the two groups. Most positive allergens were to *Dermatophagoides pteronyssinus or Dermatophagoides farina* in both groups.

**Table 1:** Comparison of inflammatory parameters (geometric mean and 95% CI) of the PAR alone group and PAR with asthma group (N = 240)

	PAR (N = 113)	95% CI	PAR with asthma (N = 127)	95% CI	*p-value*
Total IgE (IU/mL)	82.43	63.48-107.03	68.40	53.47–87.48	0.71
Eosinophils (%)	2.40	2.12–2.72	2.30	2.05–2.58	0.18
IL-1ß (pg/ml)	0.53	0.41–0.67	0.79	0.57–1.11	0.05
CCL24 (pg/ml)	196.21	162.28-237.24	236.54	199.64-280.24	0.007
iNOS (ng/ml)	14.42	10.36–20.09	56.32	45.18–70.20	< 0.001
E-cadherin(pg/ml)	102.78	97.06-108.82	89.20	79.64–99.91	0.03
Vimentin (pg/ml)	392.17	357.33–430.40	433.63	346.44-542.77	0.25

*PAR* perennial allergic rhinitis, *IL* interleukin, *CCL* C–C motif chemokine ligand, *iNOS* inducible nitric oxide synthase, *CI* confidence interval

### Independent risk factors for the development of comorbidity of PAR and asthma

Table 2 presents the univariate analysis of factors, including clinical factors and biomarker expression, regarding the presence of comorbidity of PAR and asthma. In the univariate analysis, IL-1β (> 10.84 pg/ml), iNOS (> 43.63 pg/ml), and vimentin (> 486.0 pg/ml) were associated with the development of comorbidity of PAR and asthma (*p =* 0.003, 0.038 and 0.005, respectively). The history of allergic disease of the parents or other clinical records, such as birth type and a history of general anesthesia, did not contribute to the comorbidity of PAR and asthma.

**Table 2:** Univariate analysis to determine factors related to comorbidity of PAR and asthma (N = 240)

Variables	AR (N = 113)	Comorbidity (N = 127)	*P*-value
No paternal history of AR	91	90	0.056
Paternal history of AR	22	37	
No Maternal history of AR	88	88	0.087
Maternal history of AR	25	39	
IL-1β (≤ 10.84 pg/ml)	71	55	0.003
IL-1β (> 10.84 pg/ml)	42	72	
CCL24 (≤ 308.0 pg/ml)	59	71	0.329
CCL24 (> 308.0 pg/ml)	54	56	
iNOS (≤ 43.63 pg/ml)	61	3	0.038
iNOS (> 43.63 pg/ml)	52	74	
E-cadherin (≤ 93.39 pg/ml)	61	57	0.101
E-cadherin (> 93.39 pg/ml)	52	70	
Vimentin (≤ 486.0 pg/ml)	45	73	0.005
Vimentin (> 486.0 pg/ml)	68	54	

*PAR* perennial allergic rhinitis, *IL* interleukin, *CCL* C–C motif chemokine ligand, *iNOS* inducible nitric oxide synthase

Multivariate analysis revealed that elevated IL-1β (5.6-fold increase in risk) and high expression of iNOS and vimentin (6.3 and 7.9-fold increase in risk) were significant risk factors for the development of comorbidity of PAR and asthma (Table 3).

**Table 3:** Multivariate analysis to determine factors related to comorbidity of PAR and asthma

pg/ml	95% CI		OR	*P*-value
IL-1β > 10.84	0.649	0.275	5.595	0.012
iNOS > 43.63	0.700	0.279	6.287	0.026
Vimentin > 486	0.615	0.279	4.891	0.027

*IL* interleukin, *iNOS* inducible nitric oxide synthase, *CI* confidence interval, *OR* odds ratio

### Independent risk factors for decreased lung function in PAR

We categorized patients into either the ‘normal lung function’ group or the ‘decreased lung function’ group based on the results of their pulmonary function tests. Decreased lung function was defined as a reduced FEV1 in conjunction with an FEV1/FVC ratio that is below the lower limit of normal (it is usually > 0.8 in children). We compared the biomarker expression level using geometric mean. In the normal PFT (n = 126) and decreased lung function (n = 113), IL-1β, iNOS, and vimentin were elevated in the decreased lung function group, significantly (Table 4). In addition, we investigated the risk factors for decreased lung function incorporating the results of chronic airway inflammation or remodeling. Table 5 presents a univariate analysis of risk factors, including clinical factors and biomarker expression for decreased lung function. In univariate analysis, IL-1β (> 10.84 pg/ml), iNOS (> 43.63 pg/ml), and vimentin (> 486.0 pg/ml) were associated with decreased lung function *(p =* 0.027, < 0.001 and 0.020, respectively).

**Table 4:** Expression of biomarkers (geometric mean and 95% CI) in the groups of normal lung function and decreased lung function (n = 239)

	Normal PFT (N = 126)	95% CI	Decreased PFT (N = 113)	95% CI	*p-value*
Total IgE (IU/mL)	71.26	53.45-95.00	76.54	60.6-96.66	0.31
Eosinophils (%)	2.18	1.92–2.46	2.46	2.19–2.75	0.62
IL-1ß (pg/ml)	0.51	0.36–0.72	0.46	0.59–0.98	0.05
CCL24 (pg/ml)	198.22	167.20-234.58	240.23	197.54-292.16	0.15
iNOS(ng/ml)	15.54	10.78–22.37	38.39	29.55–49.87	< 0.001
E-cadherin(pg/ml)	101.80	95.94-108.09	106.8	87.96–108.00	0.36
Vimentin (pg/ml)	384.67	345.47-428.34	427.7	396.46-495.29	0.05

*PFT* pulmonary function test, *IL* interleukin, *CCL* C–C motif chemokine ligand, *iNOS* inducible nitric oxide synthase *CI* confidence interval

**Table 5:** Univariate analysis to determine factors related to decreased lung function (n = 239) *IL* interleukin, *CCL* C–C motif chemokine ligand, *iNOS* inducible nitric oxide synthase

Variables	AR (N = 126)	Comorbidity (N = 113)	*P*-value
No paternal history of AR	95	85	0.100
Paternal history of AR	31	28	
No Maternal history of AR	92	84	0.087
Maternal history of AR	34	29	
IL-1β (≤ 10.84 pg/ml)	57	68	0.027
IL-1β (> 10.84 pg/ml)	68	45	
CCL24 (≤ 308.0 pg/ml)	67	62	0.797
CCL24 (> 308.0 pg/ml)	59	51	
iNOS (≤ 43.63 pg/ml)	74	31	< 0.001
iNOS (> 43.63 pg/ml)	52	82	
E-cadherin (≤ 93.39 pg/ml)	67	50	0.195
E-cadherin (> 93.39 pg/ml)	59	63	
Vimentin (≤ 486.0 pg/ml)	53	65	0.020
Vimentin (> 486.0 pg/ml)	73	48	

Multivariate analysis revealed that elevated IL-1β (7.8-fold increase in risk) and high expression of iNOS and vimentin (22.3 and 6.7-fold increase in risk) were significant risk factors for the development of decreased lung function (Table 6). Furthermore, elevated iNOS was the strongest risk factor for comorbidity and decreased lung function.

**Table 6:** Multivariate analysis to determine factors related to decreased lung function

pg/ml	95% CI		OR	*P*-value
IL-1β > 10.84	−0.933	0.289	7.845	0.005
iNOS > 43.63	0.288	1.359	22.333	< 0.001
Vimentin > 486	−0.707	0.287	6.742	0.009

*IL* interleukin, *iNOS* inducible nitric oxide synthase, *CI* confidence interval, *OR* odds ratio

### Relationship between IL-1β and other biomarkers

To further investigate the mechanism of IL-1β inducing iNOS and EMT, correlation analysis was used to detect the expression of candidate signaling pathway-related proteins. A regression analysis showed that the IL-1β was associated with increased vimentin and CCL-24 expression and decreased E-cadherin in children with AR (*p* = 0.001) (Table 7). However, IL-1β did not correlate with iNOS (*p = 0.094*). Therefore, we analyzed the correlation of iNOS and other inflammatory biomarkers and found that the expression of iNOS was correlated with the expression of CCL-24 (Table 8). Thus, IL-1β or iNOS can independently induce comorbidity or decrease of lung function in AR through activation of CCL-24 or vimentin.

**Table 7:** Multiple linear regression between IL-1β and inflammatory markers

Variables	Standardized coefficient (95% CI)	Adjusted *R*2	*P*-value
iNOS	0.108 (− 0.001–0.946)	0.008	0.094
CCL24	0.005 (0.002–0.195)	0.045	0.001
Vimentin	0.005 (-0.651–0.998)	0.096	< 0.001
E-cadherin	−0.001(− 0.015–2.062)	0.019	0.018

*IL* interleukin, *iNOS* inducible nitric oxide synthase, *CI* confidence interval, *CCL* C–C motif chemokine ligand

**Table 8:** Multiple linear regression between iNOS and inflammatory markers

Variables	Standardized coefficient (95% CI)	Adjusted *R*2	*P*-value
CCL24	0.241 (0.032–0.100)	0.058	< 0.001
Vimentin	0.011(− 0.025–0.029)	−0.004	0.866
E-cadherin	−0.105 (− 0.129–0.116)	−0.004	0.917

*iNOS* inducible nitric oxide synthase, *CI* confidence interval, *CCL* C–C motif chemokine ligand

## Discussion

Previously, IL-1β level in the baseline sputum was reported to be a predicting marker for poor improvement in lung function in neutrophilic asthmatics. The potential mechanism may be related to IL-1β augmenting TGF-β1-induced steroid-resistant EMT pathways [15]. In this study, we observed that children with higher IL-1β levels faced increased risks of AR and asthma comorbidity and decreased lung function. And vimentin, a marker of airway remodeling, was identified as a risk factor for both comorbidity and decreased lung function. Furthermore, elevated IL-1β was associated with increased vimentin expression in children with perennial AR, suggesting that IL-1β can trigger airway remodeling as comorbidity develops through vimentin activation. Yet, most inhibitors targeting IL-1β lack specificity and efficacy, indicating a potential need for site-specific therapies to suppress inflammation and airway remodeling more effectively [22, 23]. Thus, the development of targeted site-specific therapies may be more beneficial in suppressing inflammation and airway remodeling.

Biologically, NO is derived from L arginine and oxygen in a reaction catalyzed by NO synthases. The iNOS makes the most significant amount of NO, and iNOS is usually induced by inflammatory or immunological stimuli such as cytokines. And iNOS is expressed in airway epithelial cells in asthmatic patients and is predominantly expressed in macrophages and epithelial cells during airway inflammation [24]. NO levels increase in FENO in asthmatic patients is mainly induced by increase of expression and activity of the iNOS enzyme [25]. However, the pathophysiological role of increased iNOS in AR, particularly in children, remains unclear. Our findings reveal that iNOS can be a risk factor of comorbidity of PAR and asthma and decreasing lung function, evidenced by its correlation with CCL-24.

Interestingly, the IL-1β was not directly correlated with iNOS. These results suggest that IL-1β and iNOS can independently induce comorbidity or decrease lung function in children with perennial AR through association with airway remodeling biomarkers. Although these results provide valuable insights, our study did not include experimental data, leaving room for further exploration of the candidates studied. The identified potential biomarkers shift the focus towards discovering diagnostic and therapeutic tools for allergic disease comorbidity. Despite these limitations, our findings are significant, demonstrating oxidative and inflammatory changes in children with allergic comorbidity.

This study identified several factors associated with comorbidity and decreased lung function in children with perennial AR. Our findings add new information to identify children with PAR at an increased risk for comorbidity.

## Conclusions

This study indicates that alongside IL-1β, increased serum iNOS levels could serve as biomarkers for comorbidity of PAR and asthma and decreased lung function. Furthermore, our identification of these biomarkers provides an opportunity to uncover previously unknown mechanisms and to design effective treatments for the comorbidity of asthma and AR, as well as severe asthma.

## Data Availability

The datasets used and/or analyzed during the current study are available from the corresponding author upon reasonable request.

## Abbreviations

IL: interleukin
iNOS: inducible nitric oxide synthase
CCL: C–C motif chemokine ligand
OR: odds ratios
CI: confidence intervals
